# Description of a Novel Murine Model for Ileocystoplasty and Early Histologic Changes

**DOI:** 10.1100/tsw.2011.127

**Published:** 2011-07-07

**Authors:** Matthew R. Braasch, Thomas S. Griffith, Christopher S. Cooper, J. Christopher Austin

**Affiliations:** Department of Urology, University of Iowa, Iowa City, USA

**Keywords:** ileocystoplasty, urinary bladder, neurogenic bladder, carcinogenesis, bladder cancer

## Abstract

There is concern that bladder augmentation with bowel segments predisposes toward the development of carcinoma. Additionally, patients with neurogenic bladder and bladder cancer often present with advanced stage and have poor survival. Cellular hyperproliferation at the urointestinal junction (UIJ) has been implicated in this scenario. We aimed to develop a reproducible murine model of ileocystoplasty (ICP). We also performed preliminary analysis of any early histologic changes with focus on cellular proliferation at the UIJ. Fifteen 6- to 8-week-old female C57BL/6 mice underwent ICP, where a 1-cm ileal segment was used for bladder augmentation. Four sham mice underwent cystotomy and closure, and four mice did not undergo surgery. The mice were euthanized at 12 weeks postsurgery, and paraffin sections were stained for hematoxylin and eosin (H&E). Cellular proliferation was investigated using Ki-67. A novel model of ICP in mice was developed and demonstrated to be technically feasible in approximately 60 min under the operating microscope. Twelve-week postsurgical survival rates were 80% (12 of 15). The surviving mice had a similar weight gain as the sham mice. H&E sections showed thickened urothelium (six to 10 cell layers) at the UIJ, but sparse mitotic figures and no dysplastic changes. Ki-67 staining was rare in the urothelium, and showed no differences between the sham and ICP mice in the bladder or at the UIJ. We here demonstrate the first murine model of ICP. Preliminary studies did not show evidence of early hyperproliferation at the UIJ or in the bladder, but further long-term studies as well as studies with transgenic mice are warranted.

## INTRODUCTION

Intestinal segments are frequently used to augment the native bladder in patients afflicted with neurogenic bladder. This is commonly associated with spina bifida, which affects about 1/1000 to 1/2600 live births annually in the U.S.[1], as well as those with spinal cord injury or non-neuropathic afflictions (e.g., exstrophy, posterior urethral valves, non-neurogenic neurogenic bladder). Over 90% of patients with spinal dysraphism have some degree of lower urinary tract dysfunction[2]. Bladder augmentation has been an important means of providing continence and renal protection for patients who cannot be adequately managed with intermittent catheterization and anticholinergic medications. It is estimated that over 5% of spina bifida patients have undergone augmentation cystoplasty, with a mean age at surgery of 10.4 years old[3].

Evidence exists for increased risk of bladder carcinoma after surgical incorporation of bowel into the urinary tract. This has been most clearly demonstrated after ureterosigmoidostomy[4], with one series showing a carcinoma rate of 9.7%[5]. Surgical augmentation with small bowel or colon segments has an estimated long-term carcinoma rate of 1.2–5.5%[6,7,8,9]. In many cases, carcinoma afflicts young individuals, typically in the fourth or fifth decade of life. Additionally, there is a preponderance of more lethal histologic subtypes, such as squamous cell and adenocarcinoma[6,10,11]. Some have recommended yearly screening cystoscopy[7,12] and cytology[6,8], beginning no later than 5–10 years after surgery[11].

The incorporation of bowel into the urinary tract, along with chronic infection and repetitive catheterization, together serve as potential risks for the development of bladder cancer. Controversy exists regarding the degree that each contributes to carcinogenesis in this subset of patients. To date, some have shown mixed evidence of neoplastic changes in bladder-augmented rats[13,14,15,16] and canines[17]. However, a mouse model for studying bladder augmentation has not been reported. The murine model provides several advantages. In particular, mice are the most widely studied species for bladder cancer research, and there is wide availability of transgenic and knock-out strains for rigorous investigation. The aim of this study was to develop a reproducible surgical model for bladder augmentation in the mouse. In addition, we performed a preliminary analysis of the urothelial-intestinal junction at 12 weeks.

## MATERIALS AND METHODS

### Surgical Procedure

All experiments were approved by the University of Iowa Institutional Animal Care and Use Committee. Six- to 8-week-old female C57BL/6 mice were obtained from the National Cancer Institute and were housed for at least 1 week prior to the operation. Three to 5 days prior to surgery, the mice were converted from standard chow to Microstabilized Liquid Rodent Diet (Purina Mills, Gray Summit, MO). All food was then withheld 24 h before the operation, but the mice were provided *ad lib* access to water.

General anesthesia was induced using intraperitoneal ketamine (86 mg/kg)/xylazine (13 mg/kg) and the mice were provided preoperative antibiotic prophylaxis with subcutaneous cefazolin (200 mg/kg). Each mouse was placed supine and the abdomen was prepped with betadine, then rinsed with 95% ethanol. An appropriate level of anesthesia was assured by testing for heel reflex.

Ileocystoplasty (ICP) was performed on 15 mice under the operating microscope at 6-16X magnification through a 1.5-cm midline incision. Sharp dissection was used to enter the peritoneum. The cecum was identified and used as a landmark for identifying the distal small bowel. A 0.5- to 1-cm segment of ileum with the adjacent vascular pedicle was isolated. The segment was ligated proximally with a single 7-0 polyglactin suture placed circumferentially around the bowel and local vascular arcade. Other arcade vessels were tied off as necessary to prevent bleeding. The segment was incised proximally and the circumferential suture left in place to provide closure at the butt end of the bowel harvest. The distal end was then incised. The bowel contents were rinsed from the ileal segment using saline and kept clear from the field. Bowel continuity was restored in an end-to-end fashion using interrupted 7-0 polyglactin.

The bladder was bivalved in the midline. The urointestinal anastamosis was completed with the bowel in a chimney configuration using running, single-layer 7-0 polyglactin. The abdomen was closed in two layers using running 7-0 polyglactin. Bupivacaine 0.25% was infiltrated subcutaneously at the wound site at the end of the procedure to provide postoperative analgesia. The mice were observed until they had awoken from anesthesia and were ambulatory, and then they were returned to the institutional animal housing facility.

In the four sham mice, the procedure was performed as above with the exception of any bowel manipulation. Rather, a midline cystotomy was performed and the bladder was then closed in a running, single-layer fashion using 7-0 polyglactin. The four 16-week-old control mice did not undergo surgery.

The mice were provided buprenorphine 0.075 mg/kg subcutaneously every 12 h for two doses postoperatively for pain control. They were fasted for 24 h after surgery, but provided *ad lib* access to water. On postoperative day 2, diet was restarted with Microstabilized Liquid Rodent Diet (Purina Mills). Seven days postoperatively, all mice were converted to a diet of standard chow and were housed in the animal care facility for the duration of their postoperative course.

## Histology

Twelve weeks after surgery, the mice were euthanized according to protocol using an intraperitoneal injection of ketamine/xylazine followed by cervical dislocation once they had reached a level of general anesthesia. The mice were weighed before surgery and at the time of bladder harvest. The bladders were removed and fixed in zinc formalin overnight. The tissue was processed and embedded in paraffin; 5-µm sections were mounted on poly-L-lysine glass slides.

The Sakura Autostainer (Sakura Finetek, Torrance, CA) was used to produce H&E-stained sections. Paraffin-embedded sections were preheated in a paraffin oven at 60°C for 15 min. They were then placed in xylene (3 × 3 min), followed by sequential ethanol washes of 100% ethanol (2 × 3 min), 95% ethanol (1 min), and 70% ethanol (1 min). Slides were washed in double-distilled water ($ddH_2O$) for 2 min, then stained with hematoxylin for 6 min. They were then washed with $ddH_2O$ (1 min), hydrochloric acid/ethanol (5 sec), $ddH_2O$ (3 min), Scott's tap water (30 sec), $ddH_20$ (30 sec), 80% ethanol (30 sec), and then working eosin (30 sec). Slides were then rinsed with 95% ethanol (5 sec), followed by 100% ethanol (3 × 1 min), and then xylene (3 × 1 min). They were then coverslip mounted with solvent S-100.

For Ki-67 staining, bladder sections were preheated at 56°C, then deparaffinized and rehydrated with $ddH_2O$. After endogenous peroxidase blocking with 3% $H_2O_2$ in methanol for 5 min, antigen retrieval took place in prewarmed 10 mM citrate buffer (pH 6.0) at 95°C. The slides were treated with Triton X-100 0.1% (Sigma-Aldrich, St. Louis, MO) for 10 min, followed by avidin and biotin blocking with egg solution and 5% milk for 10 min each. Further blocking steps took place under serum-free media (DAKO Inc) for 1–2 h at room temperature. The slides were thoroughly rinsed with Tris-buffered saline (pH 7.2) between each step. Next, the primary antibody (monoclonal rat anti-Ki-67 antigen IgG2a, clone TEC-3 [M7249, Dako, Glostrup, Denmark]) was diluted in serum-free media to 1:100 and placed on the slides for 2 h at room temperature. The slides were thoroughly rinsed and the secondary antibody (Rat HRP-Polymer kit [RT517H, Biocare Medical, Concord, CA]) was applied. The slides were stained with the Chromogen-DAB kit (Vector Labs, Burlingame, CA) for 6 min, then rinsed, dehydrated, and the coverslip mounted with solvent S-100.

Using the Microsoft Excel data analysis tool, continuous data were compared using analysis of variance. Differences were considered significant at $p < 0.05$.

## RESULTS

At 12 weeks postoperatively, 12 of 15 (80%) of the ICP mice were surviving. All four sham surgery mice survived 12 weeks. The four 16-week-old control mice (no surgery) were also euthanized and the bladders harvested. Mortality in the augmented mice was due to small bowel obstruction in two mice and anastomotic bowel leak in one. On average, mortality occurred 6.7 days postoperatively. The ICP mice had a similar mean weight gain (3.6 ± 2.7 g) vs. the sham mice (4.7 ± 2.4 g) between the time of surgery and euthanasia ($p = 0.27$). No bladder stones were observed in either cohort on gross examination.

Bladder sections were evaluated after standard H&E staining and showed no evidence of malignancy in any of the ICP, sham, or control mice. Notably, the urothelium was thickened at the region of the urointestinal junction (UIJ) of all augmented mice (Fig. 1). While the normal bladder mucosa of the control mice and the sham surgery mice was three to five cell layers thick, the urothelium at the bladder-bowel junction was consistently greater than 10 cell layers. This was not observed elsewhere along the mucosa of augmented bladders. However, mitotic figures were very rare and there was no difference in comparison with other regions of the bladder. In addition, no inflammatory infiltrate was observed at the UIJ.

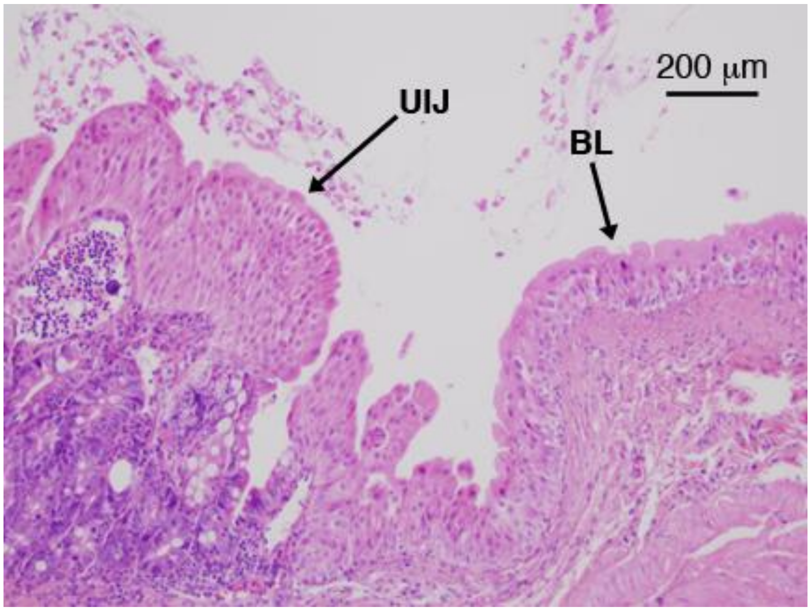

**FIGURE 1.** H&E-stained section of the ICP bladder demonstrating thickening of the urothelium overlying the bowel mucosa at the urointestinal junction (UIJ) relative to bladder (BL).

Ki-67 staining was generally noted in less than five urothelial cells/HPF and showed no differences between the ICP and sham mice. Furthermore, there was no difference in staining along the bladder, bowel mucosa, or at the UIJ (Fig. 2). The intestinal crypt cells, which have high cellular turnover and stain positively for Ki-67, served as an internal positive control.

## DISCUSSION

Concern about the development of bladder cancer in young adults following bladder augmentation for congenital bladder dysfunction was recently reported by Soergel et al.[7]. Following this and other reports, concerns about bladder cancer have led to active screening of young adults for bladder cancer after a certain age or number of years since bladder augmentation. The molecular and genetic events leading to malignancy in patients with bladder augmentation have been difficult to understand because of the long latent interval and its infrequent occurrence. Reported carcinoma rates have been 1.2–5.5%[6,7,8,9].

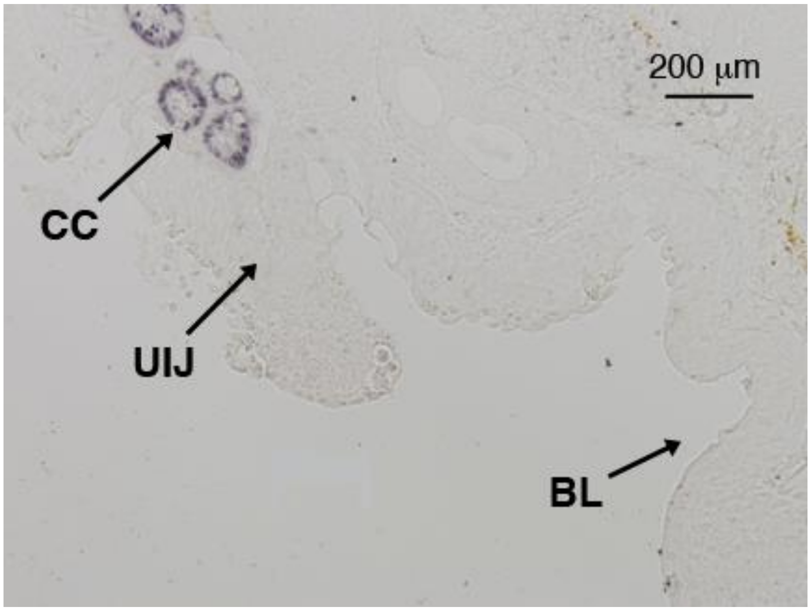

**FIGURE 2**. Ki-67–stained section of the ICP bladder demonstrating sparse Ki-67 staining of the urothelium at the urointestinal junction (UIJ) and bladder (BL). Intestinal crypt cells (CC) stain positively for Ki-67.

There is controversy as to the degree that bladder augmentation itself may contribute to carcinogenesis or whether additional confounding factors, such as trauma due to intermittent or indwelling catheterization, chronic inflammation due to urinary tract infection, or factors such as nitrosamines related to chronic bacteruria, play a larger role. It is likely a complex interaction of different factors. For example, the relative risk of bladder cancer is 12.8 times greater in spinal cord–injured patients with a history of chronic urinary catheterization compared to those without[18]. Even in cases of clean intermittent catheterization alone, there are reports of high-grade urothelial or squamous cell cancer developing in young people[19,20]. Patients with a history of three or more urinary tract infections have double the lifetime relative risk for developing bladder cancer compared to those without any infections and nearly five times the risk for developing squamous cell subtype[21]. While urine cultures were not obtained in the present study, this would be interesting for further investigations.

We previously reported a single institution series of eight spina bifida patients who developed bladder cancer in adulthood; however, only one had a history of bladder augmentation (ICP)[22]. Review of previously published cases of bladder cancer in spina bifida patients revealed that only 37% had previously undergone augmentation. To further investigate the etiology of these tumors, we developed a murine model of bladder augmentation.

Experimental models of ICP have shown a predisposition to proliferative changes and neoplasia in bladder-augmented rats[13,14] and canines[17]. With longer-term follow-up (12–21 months), bladder stones and urothelial cancer were observed in a small number of ICP and colocystoplasty rats in these studies, but not in sham surgery controls. Six- to 8-week-old mice have been the subject of much research on chemically induced bladder tumors[23], but not in augmented bladders. This is equivalent to the adolescent period in the mouse life span. Here we have demonstrated a surgical model for murine ICP with good survival. Mice in both experimental groups showed equivalent weight gain, indicating no severe metabolic or nutritional effects from the ICP. However, serum electrolytes were not evaluated here.

The UIJ showed a thickened urothelial cell layer covering the glandular epithelium of the bowel segment. Canine experiments by Gitlin et al. demonstrated both uroplakin and mucin staining of the urothelial cells covering the glandular epithelium, indicating that these cells had undergone genotypic changes consistent with enteric epithelium[17]. It was postulated that this may have implications for bladder carcinogenesis.

In the ICP mice, however, Ki-67 staining did not show early cellular hyperproliferation at the UIJ or elsewhere in the bladder. With this model, future studies may define the relative contribution of ICP vs. chronic inflammation from other sources. Ideally, the project design will be expanded to include several cohorts comparing augmentation alone to augmentation with exposure to foreign bodies and chronic bacteruria. The mouse model is important because of multiple transgenic and knock-out strains available to accelerate tumor growth. There are multiple other cell proliferation and inflammatory markers available for immunohistochemistry that could be applied to this model in the future (PCNA, COX-2, p53, survivin, among others). Some limitations of this study include the small sample size and relatively short-term histologic changes. Longer-term studies with larger cohorts are needed in order to identify chronic changes in the bladder that may predispose to carcinogenesis in ICP patients.

## CONCLUSIONS

We describe the first successful model for murine ICP. Preliminary evaluation of changes at 12 weeks in ICP mice are mild thickening of the bladder epithelium at the UIJ, but no evidence of dysplasia or increased cell proliferation in the bladder or at the UIJ. This model has many potential applications toward understanding the potential origin of carcinogenesis after incorporation of bowel segments into the urinary tract.
